# Dietary Interventions for Adults with Type 1 Diabetes: Clinical Outcomes, Guideline Alignment, and Research Gaps—A Scoping Review

**DOI:** 10.3390/nu17213349

**Published:** 2025-10-24

**Authors:** Beata Małgorzata Sperkowska, Agnieszka Chrustek, Anna Gryn-Rynko, Anna Proszowska

**Affiliations:** 1 Department of Toxicology and Bromatology, Faculty of Pharmacy, Ludwik Rydygier Collegium Medicum in Bydgoszcz, Nicolaus Copernicus University in Toruń, A. Jurasza 2 Street, 85-089 Bydgoszcz, Poland; annaproszowska@cm.umk.pl; 2 Department of Pathobiochemistry and Clinical Chemistry, Faculty of Pharmacy, Collegium Medicum in Bydgoszcz, Nicolaus Copernicus University in Toruń, M. Curie-Skłodowskiej 9, 85-094 Bydgoszcz, Poland; a.chrustek@cm.umk.pl; 3 Department of Pharmacology and Toxicology, Faculty of Medicine, University of Warmia and Mazury, Warszawska 30, 10-082 Olsztyn, Poland; anna.gryn-rynko@uwm.edu.pl

**Keywords:** type 1 diabetes, T1D, dietary interventions, low-carbohydrate diet, carbohydrate counting, Mediterranean diet, glycemic control, quality of life, psychosocial factors, dietary guidelines

## Abstract

Background/Objectives: Medical nutrition therapy (MNT) is a crucial component of type 1 diabetes (T1D) management; however, the effectiveness of specific dietary approaches in adults remains unclear due to variations in study design, terminology, and reported outcomes. This scoping review summarizes evidence published between 2015 and 2025 on dietary interventions in adults with T1D, focusing on metabolic and psychosocial outcomes and adherence to international nutritional guidelines. Methods: We searched PubMed, Web of Science, Scopus, and Google Scholar, following the PRISMA-ScR recommendations, to identify observational studies, randomized clinical trials (RCTs), and guidelines involving adults (≥18 years) with T1D. Extracted data included metabolic outcomes (glycated hemoglobin A1c (HbA1c), glycemic variability (GV), insulin dose (ID), lipids, blood pressure, body weight, and others), as well as psychosocial indicators (i.e., quality of life, diabetes-related stress, and fear of hypoglycemia). Results: In total, 41 studies met the inclusion criteria, comprising 18 observational, 14 randomized, and 9 studies that evaluated psychosocial aspects. A low-carbohydrate diet (LCD) reduced HbA1c by 0.3–0.9% and total ID by approximately 15–20% without increasing the incidence of severe hypoglycemia. A low-fat vegan diet and structured carbohydrate counting (CC) programs also improved glycemic and lipid profiles. The Mediterranean diet (MedDiet) and plant-based diet mainly improved diet quality and well-being. The results showed an association between better metabolic control and lower carbohydrate (CHO) intake, as well as higher intakes of fiber and protein. In contrast, a Western diet and high intake of sweets were linked to poorer outcomes. Conclusions: Combining an LCD with education, CC, and modern diabetes technology provides the most consistent benefits for adults with type 1 diabetes (T1D adults). The MedDiet and plant-based diet support diet quality and psychosocial well-being, although current evidence remains limited, primarily due to small sample sizes and short follow-up periods.

## 1. Introduction

Diabetes mellitus (DM) is a heterogeneous metabolic disorder affecting people of all ages worldwide [1,2,3]. According to the 11th IDF Atlas, the global number of people with DM is expected to rise by 45% from 2024 to 2050, reaching 853 million, with the fastest growth in Africa and Asia, and the slowest in Europe [1]. Although T1D represents only a small proportion of cases (approx. 9.5 million adults vs. >500 million with type 2 diabetes), it is marked by high clinical complexity [3,4]. Adults with T1D commonly experience recurrent episodes of hypo- and hyperglycemia, predisposing them to acute and chronic complications that impair quality of life (QoL) [5,6]. This growing burden highlights the importance of optimizing treatment strategies for T1D adults.

Insulin therapy, delivered via multiple daily injections, pumps, or hybrid closed-loop systems, remains the cornerstone of treatment. However, optimal outcomes also require non-pharmacological strategies, including MNT, physical activity (PA), psychological support, and structured care. Major diabetes organizations, the American Diabetes Association (ADA), the International Diabetes Federation (IDF), and the European Association for the Study of Diabetes (EASD), emphasize self-monitoring, continuous glucose monitoring (CGM), and dietary guidance focusing on individualized macronutrient distribution, limited added sugars, and adequate fiber [5,7,8,9,10].

Beyond technological advances, dietary strategies remain central to effective disease management. Over recent decades, various nutritional approaches, including LCD, low-glycemic index, ketogenic diet, MedDiet, DASH, and plant-based, have been investigated in T1D adults [4,11,12,13,14,15,16,17,18,19,20,21].

Other strategies, including CC [22,23,24,25,26,27,28], protein adjustment [22,29,30], or coeliac-specific modifications [31,32,33], target more specific needs of this population [33,34]. While some studies suggest benefits for glycaemic control and cardiometabolic outcomes, the evidence remains inconsistent [32,35,36,37]. Emerging approaches, such as intermittent fasting (IF), also require further validation [38,39,40,41,42,43].

Current research is limited by small sample sizes, short follow-up periods, heterogeneous outcomes [13,44,45], and cultural variability, which reduces generalizability [13,44,45,46]. Beyond methodological limitations, psychosocial factors such as fear of hypoglycemia (FoH), depression, stress, and disordered eating further complicate adherence [47,48,49,50,51]. Depressive symptoms are present in 31–38% of adults with T1D in the current era. This figure is 30.7% in the multinational International Diabetes Management Practices Study (IDMPS) [52] and 38.3% in a national Kuwaiti cohort [53]. Depressive symptoms are consistently associated with poorer metabolic control, including higher HbA1c and less favorable glycemic profiles [54]. Although education, peer support, and digital tools may enhance self-management, they can also contribute to “technological stress” [55,56,57].

MNT provided by qualified dietitians remains central to care, with individualized interventions shown to lower the level of HbA1c by approximately 1–2% and improve cardiometabolic health [8,58,59,60,61,62,63]. However, restrictive regimens such as very low-carbohydrate (VLCD) diets remain controversial in daily practice for patients with T1D [39,64,65,66,67].

To address this gap, the present scoping review aims to (i) map current evidence, (ii) identify methodological limitations, and (iii) highlight priorities for future clinical practice and research.

## 2. Materials and Methods

### 2.1. Protocol and Reporting Guidelines

This scoping review was conducted in accordance with the PRISMA-ScR (Preferred Reporting Items for Systematic Reviews and Meta-Analyses extension for Scoping Reviews) guidelines [68]. We prepared the flow diagram using the PRISMA 2020 template [69]. The study protocol was developed a priori, specifying eligibility criteria, search strategy, and methods for study selection and data extraction.

### 2.2. Data Sources and Search Strategies

A period of ten years was selected to capture the most up-to-date evidence on dietary interventions in adults with T1D, reflecting major technological advances (e.g., CGM) and updates to nutritional recommendations.

The search strategy was structured around three main conceptual areas: (1) population—T1D adults; (2) dietary interventions, including the MedDiet, DASH, ketogenic diet, LCD, low-glycemic index, vegetarian, vegan, plant-based, CC, fasting, high-fiber, and gluten-free diets (GFD), and (3) clinical and psychosocial outcomes, such as HbA1c, time in range (TIR), GV, hypoglycemia (HypoG), total daily insulin dose (TDI), lipid profile, body mass index (BMI), diabetes complications, quality of life (QoL), adherence, diabetes distress (DD), depression, and FoH.

Search terms, logical operators, and MeSH descriptors were adapted for each database. Additionally, the reference lists of included publications were manually screened, and citation tracking (snowballing) was performed to ensure comprehensive literature coverage.

### 2.3. Dietary Exposure Definitions and Carbohydrate Standardization

To ensure clarity and consistency in terminology, detailed operational definitions of dietary exposures (comparators, carbohydrate thresholds in grams per day and/or percentage of energy, assessment/adherence tools, and standardization rules) are provided in the Supplementary Materials (Tables S1 and S2). In the main text, we retain author-reported categories without converting grams to EI or inputting total energy intake.

### 2.4. Inclusion and Exclusion Criteria

We defined the subject area using the PCC (Population—Concept—Context) framework (Table 1). We included studies that enrolled adults (≥18 years) with confirmed T1D and evaluated the effects of dietary interventions or habitual eating patterns on clinical, metabolic, or psychosocial outcomes. Eligible sources comprised observational studies, RCTs, and scientific society guidelines published in English within the predefined search period.

We excluded studies involving children or adolescents and those focused on other types of diabetes (type 2, gestational, or latent autoimmune diabetes (LADA) in adults, which is a heterogeneous phenotype with a slower rate of β-cell decline and distinct insulin and dietary needs compared with classical T1D). In analyses involving mixed populations, we only included data when the authors provided distinct outcome data for T1D adults. Only in exceptional cases, when a non-T1D subgroup was a small minority and could not be separated analytically, did we retain the study and clearly indicate the mixed population. Thus, researchers retained one study involving a small group of individuals with LADA (approximately 10%) without separate estimates for T1D and labeled it as T1D/LADA. They conducted sensitivity checks and confirmed that its inclusion did not change the direction of effects across different outcomes.

We also excluded study protocols without outcome data, conference abstracts, commentaries, editorials, and manuscripts that were incomplete or non–peer–reviewed. We did not include studies assessing acute or single-meal postprandial effects (lasting ≤ 24 h). We considered only interventions lasting at least 1 week or studies evaluating habitual dietary patterns.

### 2.5. Selection and Data Extraction

We imported all search results into Mendeley Reference Manager, and A.G.-R. curated the dataset for screening and extraction. Two independent reviewers (A.G.-R. and A.P.) screened the titles and abstracts according to predefined inclusion and exclusion criteria, followed by an assessment of the full-text publications.

Any disagreements were resolved through discussion and, when necessary, by consulting senior reviewers (B.M.S. and A.C.).

From each included study, the following data were extracted and analyzed: author, year of publication, country; population characteristics (sample size, percentage of females, mean age, and range where applicable, and diabetes duration); description of the dietary intervention; comparison group (if applicable); and clinical, metabolic, psychosocial, and behavioral outcomes.

The primary indicator of intervention effectiveness was HbA1c, with additional metabolic and psychosocial outcomes considered secondarily when reported. Information on adherence to dietary recommendations, dropout rates, and the geographical, cultural, and healthcare system context was also recorded.

We entered the extracted data into a standardized Excel spreadsheet to ensure consistency and to facilitate subsequent classification by study type and year of publication.

### 2.6. Data Synthesis

The collected data were presented in both descriptive and tabular formats. Given the limited number of studies, particularly RCTs addressing clinical and psychosocial outcomes, the results were first grouped by study design type and then arranged chronologically by year of publication.

Four summary tables were developed: Table 2 presents the impact of nutritional interventions on HbA1c and other metabolic indicators for observational studies; Table 3 summarizes the corresponding results in RCTs; Table 4 illustrates the relationships between nutritional and psychosocial factors, and Table 5 compares current and historical dietary recommendations for T1D adults.

In accordance with scoping review methodology, no quantitative meta-analysis or formal risk of bias assessment was performed. The conclusions were presented descriptively, and the tables highlight the key patterns, research gaps, and regional differences identified across the included studies.

## 3. Results

A total of 4437 records were identified in databases and 47 from other sources. After removing 2054 duplicates, 2383 records were screened; 2049 were excluded at the title/abstract stage. A total of 334 articles were eligible for full-text analysis, of which 301 were excluded according to the criteria. Ultimately, 41 studies were included: 18 observational, 14 randomized controlled trials (RCTs), and 9 on psychosocial determinants.

**Figure 1:**
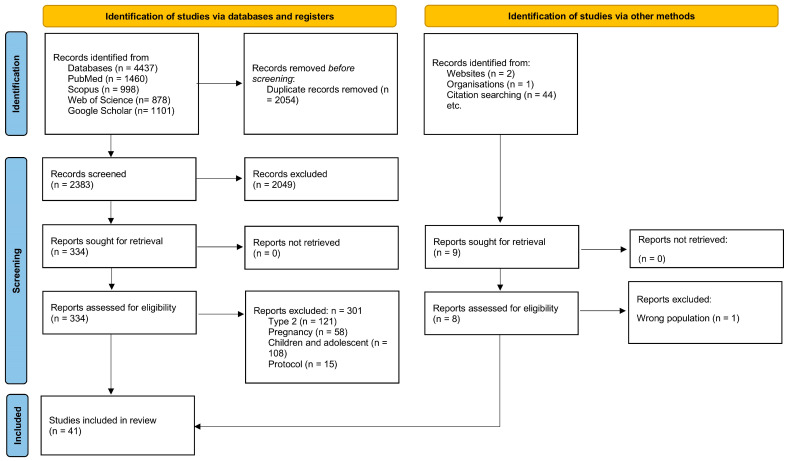
PRISMA 2020 flow diagram for identification, screening, eligibility, and inclusion in this scoping review of dietary interventions in adults with type 1 diabetes (*n* = 41 total: 18 observational, 14 RCTs, 9 psychosocial) [69].

Figure 1 (PRISMA-ScR) presents the selection process, while Section 3.1.1, Section 3.1.2, Section 3.1.3 and Table 2, Table 3 and Table 4 present the characteristics of the studies [69].

### 3.1. Study Characteristics

The included studies were published between 2015 and 2025 and originate mainly from Europe and North America, with a few publications from South America and Asia; regional details are listed in Section 3.1 and Table 2, Table 3 and Table 4.

The studies summarized in this review included a total of approximately 12,860 adult participants with type 1 diabetes, comprising 8682 from observational analyses, 648 from randomized controlled trials, and 3532 from psychosocial studies. The review included only data from the type 1 diabetes subgroup in studies involving mixed populations.

Participants ranged in age from 18 to 79 years, and their duration of diabetes ranged from 1 year to more than 30 years. Several studies included mixed populations of individuals with varying ages [70,71], types of diabetes T1D and T2D [72], and LADA population (9.5%) [73]; however, only data related to adult participants with T1D were analyzed in this scoping review.

Although the predominance of small, short-duration crossover interventions limits the generalizability of findings, it underscores the need for larger and longer parallel RCTs to strengthen the evidence base for dietary strategies in adults with T1D.

#### 3.1.1. Dietary Interventions in Observational Studies

In these analyses eighteen observational studies were included: 13 cross-sectional, 2 prospective/retrospective, 2 longitudinal, and 1 case–control. In the observational set (*n* = 18), individual study samples ranged from 36 to 1874 adults with T1D, and reported ages spanned 18–79 years (Table 2). The most frequently examined factors were adherence to the MedDiet (*n* = 5), and dietary patterns (including ‘Western’, ‘traditional’, and ‘semi-healthy’), as well as CC practices and LC intake. Self-reporting tools (FFQ, diaries, 24-h interviews) and pattern analysis methods (e.g., RRR/factorization) were mainly used. The range of outcomes included HbA1c, CGM metrics (TIR/GV), lipid profile, blood pressure, weight/waist circumference, and selected complication indicators. Prospective and retrospective studies investigated the relationship between macronutrient intake and glycemic control (GC) [30,74].

Moreover, the longitudinal studies examined dietary pattern adherence, including Mediterranean-style and DASH diets, as well as food-based patterns such as ‘baked desserts’ and convenience foods [75,76]. In contrast, the case–control study evaluated intake of total fat, monounsaturated fatty acids, oleic acid, vitamin E, and complex carbohydrates in relation to diabetic retinopathy [77].

The non-randomized studies included in this review encompassed a broad geographical distribution, covering regions across Europe: Finland (*n* = 3), Spain (*n* = 2), Croatia, Poland, Italy, and North America: Canada (*n* = 2), USA (*n* = 3), South America: Brazil (*n* = 2), and Asia: China, and Iran.

**Table 2:** Summary of observational and intervention studies on dietary interventions and nutritional patterns in T1D adults (*n* = 18).

Author/(Year)/Country/[Ref]	Population	T1D Duration	Dietary Pattern	Dietary Assessment Method	Outcomes Measured	Main Results
Cross-Sectional Studies (*n* = 13)						
Gingras et al. (2015), Canada [78]	Ns/e = 118; F = 52%; 23.1 * yrs	T1D (y) 23 * NR	MedDiet vs. Canadian recommendation	MedDiet Score (0–44)	HbA1c, lipids, BMI, WC, %fat, BP, eGDR	Higher MedDiet score: HbA1c and lipids ↔, ↓ BMI, ↓ WC, ↓ %fat, ↓ SBP, ↓DBP, ↑ eGDR
Jaacks et al. (2016) China [79]	Ne = 99; F= 45%; 43.6 (28.4–55.0) yrs	T1D (y) 7.8 (4.8–17.8)	RRR-derived dietary patterns: Pattern 1—low wheat & high-fat cakes, high beans & pickled vegetables; Pattern 2—low high-fat cakes/nuts/fish/tea-coffee, high rice & eggs	Three R24W (2 weekdays + 1 weekend) with FR; RRR on 20 food groups	HbA1c, LDL-c	Pattern 1: Highest vs. Lowest tertile → HbA1c + 1% (≈11 mmol/mol) and LDL-C + 0.36 mmol/L (*p* < 0.05, adjusted for age & income). Pattern 2: ns association with HbA1c or LDL-c
Ahola et al. (2018) Finland [80]	Ns = 1429; Ne = 1040 F = 54.5%; 47 * yrs	T1D (y) NR	Self-reported adh. SpD (36.6% N)—lactose-free 17.1%, protein restriction 10%, vegetarian 7%, gluten-free 5.6%, multiple other SpD	Validated diet questionnaire (SpD adh.) + FFQ for dietary patterns + 3-day food records	HbA1c, BMI, BP, serum lipids, eGFR	SpD adherents were mostly women, older, with longer T1D duration & more complications; mean HbA1c ~64–67 mmol/mol (8.0–8.3%) with no improvement; fiber intake < rec, vit D, folate & Fe often inadequate, esp. in lactose- & gluten-free diets.
Ahola et al. (2019) Finland [81]	Ns = 1000 Ne = 992; F = 58%; 47 yrs * (range not specified	T1D (y) NR	HD: macronutrients (%EI) & fiber (g/MJ)	Two validated 3-day FR (3–6 days total)	HbA1c, lipids	higher fiber (g/MJ) → lower mean SMBG (β = −0.428; 95% CI −0.624 to −0.231; *p* < 0.001); higher CHO, alcohol, MUFA → ↑ CV; substitution models: protein ↔ ↓ CV vs. CHO/fat/alcohol; fat ↔ ↓ CV vs. CHO; fiber adjustment attenuated mean SMBG associations.
Ahola et al. (2019) Finland [82]	Ns/e = 1874; F = 54.0%; 48.1 ± 13.4 yrs	T1D (y) 24 (18–34)	HD and renal assessments approx. 5 days apart (median)	Validated diet questionnaire on food habits, SpD, salt reduction, counseling; 19-item FFQ; two 3-day FR	NR	EI ↓ at all CKD stages vs. normal; protein (g/kg) ↓ progressively; Na, K, Ca, P ↓ with worsening function; fiber (g/MJ) ↑ in eGFR 60–89, 30–59, and transplants but ↓ in dialysis; rye → wheat bread shift at eGFR < 30/dialysis; liquid milk and coffee ↓ in advanced CKD; salt reduction, SpD, and counseling ↑ with progression; transplant recipients partly “liberated” (↑ fiber, K, Ca, P; ↓ strict adherence).
Granado-Casas et al. (2019) Spain [83]	Ns/e = 259 F = 54.4% age 43.7 * yrs	T1D (y) 21.5 *	Comparison of dietary habits and adh. to the MedDiet and HEI between T1D and CON	Validated 101-item interviewer-administered FFQ (Spanish version; calculated aMED (0–9) and aHEI scores	NR	T1D had ↑ MedDiet & healthier pattern: MedDiet 3.7 ± 1.6 vs. 3.2 ± 1.8 (*p* = 0.009); low MedDiet (0–2): 23.2% vs. 35.4% (*p* = 0.019); HEI 40.7 ± 6.5 vs. 37.6 ± 6.2 (*p* < 0.001); consumed ↑ dairy, proc meat, fatty fish, F/V, nuts, legumes, potatoes, bread; ↓ seafood, sweets; T1D status, ↑ age, ↑ PA, rural → ↑ aMedDiet/aHEI; male sex → ↓ HEI.
Richardson et al. (2022) USA [84]	Ns/e = 563 37 (19–56) yrs	T1D (y) NR	exposure: MSDPS, habitual intake over the past 12 mo	Validated 126-item Harvard FFQ	CAC > 0 and PAT (cm^3^)	MSDPS ↔ CAC (OR ≈ 1.00, ns); ↑ MSDPS → ↓ PAT (−0.003 cm^3^/+1 MSDPS; 95% CI −0.006 to −0.0004; *p* = 0.025), attenuated after adj lipids & PA; inverse assoc sig in non-DM, not T1D; fruit, wine & meat comps ↓ PAT (−0.02 to −0.03 cm^3^/ + 1 pt; *p* ≤ 0.0001).
Zięba et al. (2022) Poland [85]	Ns/e = 48; F = 31% - HbA1c < 6.5%: 20—HbA1c ≥ 6.5%: 28 25.6 (22.2–28.3)	T1D (y) 15 * y	HD; Two groups: HbA1c ≤ 6.5% and >6.5% value. Assessed nutrient intake using self-reported 3-day 24-h dietary surveys.	Self-reported 3-day R24W. Two groups HbA1c ≤ 6.5% and >6.5%	Nutrient intake (N, K, Ca, Mg, Fe, Zn, Cu, I, Mn, vitamins A, D, E, B1, B2, B3, B6, folate, B12, C)	T1D adults using insulin pumps had insufficient intake of most nutrients/vitamins, excessive SFA and cholesterol; PUFA, sodium, niacin, and calcium intake differed by HbA1c; need for dietary education and possible vitamin D/I supplementation.
Azulay et al. (2023) Brazil (Northeast [70]	Ns/e = 152; F = 48%; Age mix age 25.1 ± 10.6 yrs range > 10 yrs	T1D (y) 13.8 ± 8.1 y	MNT: sugar restriction (19.3%), CC (15.9%), personalized advice (64.8%); adh. ≥80% in 39.5%; regular PA ≥ 3 × /wk 30.3%	Structured questionnaire on dietary type and adh.; self-report (≥80% adh. = good); HbA1c by HPLC; ancestry via 46 AIM-indel markers	HbA1c (%); GC categories (good: <7% adults; <7.5% children/adolescents; poor: >9%); hierarchical logistic regression of factors associated with good control	Dietary adh. ↑odds of good HbA1c control (adj. OR = 2.56, 95% CI 1.18–5.59, *p* = 0.016); age > 40 year (adj. OR = 4.55, *p* = 0.031) and male sex (adj. OR = 2.00, *p* = 0.047) also ↑ likelihood; difficulty avoiding sugar ↓ control (OR = 0.51, *p* = 0.049); ancestry ns; PA ↔ HbA1c.
Uliana et al. (2023) Brazil [71]	Ns/e = 173; F = 84.4%; NR (18—59) yrs 1 week per arm; 5–35 d washout	T1D (y) < 10 y of diagnosis 5.04 *, T1D (y) > 10 y 19.64 * y	CC practice characteristics (timing, method, education source)	Online self-administered questionnaire (Google Forms^®^ (Google LLC, Mountain View, CA, USA); self-reported clinical and anthropometric data	HbA1c (adequate < 7%, increased ≥ 7%), associations with CC practice, education, and diagnosis duration	Practicing CC and diabetes duration < 10 yrs were predictors of adequate HbA1c; using apps and food scales, performing CC at lunch/dinner, and learning from a nutritionist were associated with better HbA1c.
Nguyen et al. (2024) Canada [73]	Ns/e = 285 #; F = 62.9%; 48.2 * yrs N/A	NR T1D duration 25.9 ± 16.2 y	LCD score, quartiles (Q1 = 21–30 to Q4 = 0–9);	Validated R24W, single recall	HbA1c, level-2 and level-3 hypoglycemia, LDL-c, non-HDL-c, BMI, WC.	Higher proportion with HbA1c ≤ 7% in Q1 vs. Q4 (53.4% vs. 29.4%; *p* = 0.011) with adjusted OR up to 3.22 (95% CI 1.51–6.85); greater proportion never experiencing level-3 hypoglycemia in Q1 vs. Q3 (60.0% vs. 31.0%; *p* = 0.004); no differences across quartiles for level-2 hypoG frequency or lipid profile.
Shojaeian et al. (2024) Iran [86]	Ns/e = 229; F = 61.7%; single measurement	>1 year (inclusion criterion); 69% > 10 yrs of diabetes	Dietary patterns (factor analysis): western, unhealthy, traditional, semi-healthy	Validated 168-item FFQ (12 months); 23 food groups; definitions for HbA1c > 7%, FBG > 130 mg/dL, LDLc > 100 mg/dL, low eGDR (tercile 1)	HbA1c, FBG, blood lipids (TG, TC, LDL-c, HDL-c), blood pressure, BMI, WC, WHR, body fat %, eGDR (insulin resistance)	Western (T3 vs. T1): ↑ odds FBG, HbA1c > 7%, low eGDR. Unhealthy: ↑ odds LDL-c > 100, abdominal obesity. Semi-healthy: ↓ odds FBG and TC. BP and BMI/BFP ns.
Abuqwider et al. (2025) Italy [87]	Ns/e = 198; F = 49.5%; 38.8 (19–79) yrs	T1D (y) ≥ 1 y	Usual self-selected diet; exposure = serum SCFA (acetate, propionate, butyrate)	7-day weighed FR, reviewed by dietitian (with Metadieta v4.5)	HbA1c (%), CGM metrics (TIR 70–180 mg/dL, TAR > 180 mg/dL, TBR < 70 mg/dL, GMI), BMI, therapy type	Women: high serum propionate tertile → ↑ TIR (66.2 ± 12.3% vs. 56.9 ± 16.7%, *p* = 0.014), ↓ TAR (32.2 ± 12.6% vs. 41.2 ± 17.2%, *p* = 0.011), ↓ GMI (7.1 ± 0.6 vs. 7.5 ± 0.6%, *p* = 0.027) vs. low tertile (adjusted for age, BMI). No HbA1c differences across SCFA tertiles. Men: no associations between SCFA and glycemic metrics; higher acetate tertiles linked to ↑ fat, PUFA & MUFA intake (*p* ≈ 0.04).
Prospective /retrospective studies (*n* = 2)						
Gradinjan Centner et al. (2019) [30]	Ns/e = 151 T1D; F = 60.3%; 38 (18–60) yrs	T1D duration: ≥12 mo (inclusion criterion)	Habitual intake (macronutrients: PROT, CHO, fats; fiber; sugar; minerals: Zn, Se, Mg)	7-visit food diary (in-clinic and remote)	HbA1c; CGM metrics (MG, GMI, TIR, TAR, TBR), hypo events/duration.	Baseline HbA1c inversely correlated with fiber (ρ = −0.259; *p* = 0.015); at 3 mo, higher PROT intake → lower HbA1c (ρ = −0.296; *p* = 0.012) and ↑TIR (ρ = 0.249; *p* = 0.032); HbA1c improved vs. 3-mo GMI (Δ = 0.378; *p* = 0.022); scanning frequency correlated positively with PROT (ρ = 0.489; *p* < 0.001) and selenium (ρ = 0.277; *p* = 0.019), and negatively with CHO (ρ = −0.336; *p* = 0.004); ns associations between dietary variables and HypoG.
Lehmann et al. (2020) USA [74]	Ns/e = 36; F = 28%; 36.9 (23.4—50.4) yrs	T1D (y)	MDC: 166.4 g distributed over 5.7 meals/per day		TIR (70–180 mg/dL), TAR (>180 mg/dL), TBR (<70 mg/dL), MG (mg/dL), CV (%)	Lower CHO intake → ↑ TIR (77.4 ± 15.4% vs. 75.2 ± 16.7% vs. 70.4 ± 17.8%, *p* < 0.001) and ↓TAR (20.1 ± 14.7% vs. 22.0 ± 16.9% vs. 27.2 ± 18.4%, *p* < 0.001); TBR ↔ (*p* = 0.50); +10% CHO → −1.1% TIR, +1.2% TAR (*p* < 0.001).
Longitudinal studies (*n* = 2)						
Basu et al. (2021) USA [75]	Ns/e = 1257; T1DNs/e = 568; CON Ns/e = 689); (T1D) 37 ± 9 yrs.; (CON) 39 ± 9 yrs	T1D (y) 23.5 ± 8.9 l	Dietary patterns identified by PCA (“fruits, veggies, meats, cereal”, “baked desserts”, “convenience foods and alcohol”	dietary assessment: validated Harvard FFQ (1988);	HbA1c at baseline and 6-year follow-up	“Baked desserts” pattern ↑ HbA1c at baseline and year 6; SFA, animal fats, and low/no-calorie beverages ↑ HbA1c; dark-green vegetables (baseline), tomatoes, and whole grains (year 6) ↓ HbA1c (*p* < 0.05).
Richardson et al. (2023) USA [76]	Ns/e = 1255; T1DNs/e = 563; F = 57%; 37 (19–56) yrs	T1D (y) NR	High-CHO vegan (75C/15P/10F); no kcal or CHO limit; no animal products/fats; low-GI focus	Hypocaloric (−500–1000 kcal/d, BMI > 25); 60–70C/15–20P; MUFA; <7% SFA; ≤200 mg chol.	HbA1c; LDL; CAC; PAT	+1 MSDPS → −0.09 cm^3^ PAT; +1 DASH → −0.26 cm^3^ PAT; no pooled link with CAC progression; DASH ↓ CAC progression only in non-DM.
Case–control study (*n* = 1)						
Granado-Casas et al. (2018) Spain [77]	Ns/e = 243 (103 with DR, 140 without DR); approx. 44 yrs *; F= approx. 55%	T1D (y) 26 * y in DR vs. 18 y in no-DR	HD; focus on total fat, MUFAs, oleic acid, vitamin E, complex CHO	Validated FFQ (101 items, past year)	NR	Higher complex CHO ↑ DR risk; higher total fat, MUFA, oleic acid, vit. E ↓ DR risk.

Explanation of abbreviations: *—range not specified; #—Includes 9.5% of participants with LADA; aHEI—Alternate Healthy Eating Index; aMED—Alternate Mediterranean Diet Score; BP—Blood Pressure; CAC—Coronary Artery Calcification; CC—Carbohydrate Counting; CI—Confidence Interval; CON—Controls (non-diabetic control group); CV—Coefficient of Variation; DBP—Diastolic Blood Pressure; eGDR—Estimated Glucose Disposal Rate; eGFR—Estimated Glomerular Filtration Rate; EI—energy intake; F—Female (%); FFQ—Food Frequency Questionnaire; FR—Food Record; F/U—Follow-up; GC—Glycemic Control; GDR—Glucose Disposal Rate; GI—Glycemic Index; GMI—Glucose Management Indicator; HCL—Hybrid Closed-Loop (insulin delivery system); HD—Habitual Diet; HbA1c—Hemoglobin A1c; HEI—Healthy Eating Index; LDL-c—Low-Density Lipoprotein Cholesterol; MDC—Mean Daily Carbohydrate Intake; MedDiet—Mediterranean Diet; MSDPS—Mediterranean-Style Dietary Pattern Score; MNT—Medical Nutrition Therapy; MUFAs—Monounsaturated Fatty Acids; N/Ne/Ns—Number of Participants/Ended/Started; NR—Not Reported; non-HDL-c—Non–High-Density Lipoprotein Cholesterol; ns—not significant; OR—Odds Ratio; PAT—Peripheral Arterial Tonometry; PCA—Principal Component Analysis; PROT—Protein; PUFAs—Polyunsaturated Fatty Acids; RRR—Reduced Rank Regression; R24W—24-h Web-based Dietary Recall; SBP—Systolic Blood Pressure; SFAs—Saturated Fatty Acids; SCFAs—Short-Chain Fatty Acids; SD—Standard Deviation; SMBG—Self-Monitored Blood Glucose; SpD—Special Diets; T1D—Type 1 Diabetes; TAR—Time Above Range (>180 mg/dL); TBR—Time Below Range (<70 mg/dL); WC—Waist Circumference; yrs/y—Years (age or diabetes duration); ↑ increase; ↓ decrease; ↔ no change; → tendency or direction of change.

Despite methodological variability in dietary exposures, assessment tools, and outcome measures, all studies consistently explored the relationship between dietary behaviors and metabolic or clinical outcomes in T1D adults.

#### 3.1.2. Randomized Controlled Trials

This section included fourteen RCTs (parallel and crossover designs, seven studies in each case). The exception was one study by Igudesman et al. (2023), which was classified as a parallel RCT with an adaptive SMART design because participants were not re-exposed to multiple interventions in the same phase of the study [72]. Interventions mainly included LCDs, CC programs, low-fat/plant-based and Mediterranean patterns, and single intermittent fasting protocols or macronutrient composition modifications. The duration of the interventions ranged from 1 week to 12 months. For mixed-population studies, only data from T1D adults were included. The outcomes focused on HbA1c and CGM metrics; some studies reported psychosocial aspects, including QoL, fear of FoH, and treatment satisfaction.

The randomized trials focuses in this scoping review enrolled adults aged 19–79 years with T1D; however, one trial (Igudesman et al., 2023) included a mixed population of participants with T1D and T2D [72], and another (Kahleová et al., 2024) involved children, adolescents, and adults [88]. In both cases, only data pertaining to adult participants with T1D were included in this scoping review (Table 3).

These trials originated mainly from Europe (*n* = 8) and were primarily conducted in Denmark (*n* = 4), Sweden (*n* = 3), and Greece. In addition, the remaining studies originated from North America (*n* = 2), with one each conducted in Australia, New Zealand, and South Asia (India).

Across the included studies, the most frequently assessed metabolic outcomes were HbA1c, continuous glucose monitoring (CGM) metrics, lipid profile, blood pressure, BMI, and waist circumference. This overview emphasizes the diversity of metabolic endpoints and provides a clearer context for the subsequent discussion.

The next Section 3.1.3 summarizes these findings, focusing on psychosocial and behavioral outcomes associated with dietary management in T1D patients.

**Table 3:** Summary of RCTs on dietary interventions and nutritional patterns in adults with T1D.

Author/ (Year)/ Country/[Ref]	Study Design	Population T1D Duration	Intervention Diet	Control Diet	Intervention Duration	Metabolic Outcomes	Main Results
Krebs et al. (2016) New Zealand [89]	Parallel	Ns/e = 10; INT =5, 5 CON; F = NR; 44.6 * yrs; T1D (y) 21.8 *	LCD (50–75 g/d CHO) + CC	Standard diet + CC	12 wks	HbA1c, CGM (MG, MAGE), TDI insulin, BP, BMI, lipids, creatinine.	↓ HbA1c (63→55 mmol/mol, *p* < 0.05), ↓ TDI (64.4 → 44.2 U/d, *p* < 0.05); weight trend −5 kg; no changes in variability (MAGE), BP, lipids.
Hommel et al. (2017) Denmark [25]	Parallel	Ns 168 (84 ABC, 84 MC); Ne= 130 (66 ABC, 64 MC); 46.9 * yrs (ABC); 47.1 * yrs (MC); T1D (y) >20	Advanced CC + ABC	Advanced CC + MC	12 mo	HbA1c (primary), >10 mmol/L), MG, CGM (% time < 3.9, in range, CV; weight, BP	Both groups ↓ HbA1c; ABC greater: −5 mmol/mol (−0.5%) vs. −2 mmol/mol (−0.2%), *p* = 0.033; ABC users ↑ TIR (50% vs. 30%).
Ranjan et al. (2017) Denmark [64]	Crossover	Ns/e = 10 y; F = 40%; 48.0 * yrs T1D (y) 23 ± 7 y	LCD (≤50 g/d CHO, isocaloric)	HCD (≥250 g/d CHO, isocaloric)	1 wk LCD and 1 wk HCD	HbA1c; TDI; CGM: MG, % time 3.9–10, % ≤3.9, % >10 mmol/L, CV, MAGE, CONGA, HBGI, LBGI.	LCD: ↑ TIR (83% vs. 72%, *p* = 0.004), ↓ hypoglycemia (3.3% vs. 8.0%, *p* = 0.03), ↓ GV (SD 1.9 vs. 2.6 mmol/L; CV 27.7% vs. 35.4%, *p* = 0.02); MG ↔; fasting ketones, glucagon, FFAs.
Fortin et al. (2018) Canada [90]	Randomized trial	Ns/e = 28 T1D (14 MedDiet, 14 LF); 50.9 * 0.2 yrs T1D (y) 26.8 ± 15.2 y	MedDiet quality focus: olive oil, fish, legumes, nuts, and vegetables)	LFD (reduced fat, lean PROT, limited fried foods)	6 mo	HbA1c, HypoG, BMI, weight, fat mass, eGFR, BP, lipids, hs-CRP, WC (primary).	HbA1c unchanged; trend to ↑ well-being in MedDiet (+ 5.3 vs. −5.4, *p* = 0.08), Both groups ↓ WC (−3.3 cm low-fat vs. −1.5 cm MedDiet, NS); ↓ BMI and weight in both groups.
Overland et al. (2018) Australia [91]	Parallel	Ns/e = 10 (5 IF, 5 CER); 49.6 * yrs (IF); 44.1 yrs (CER); T1D (y); (IF = 24.5 (4.6–34.6) CER= 29.5 (18.0–40.8)	IF: 600 kcal/d, 2 d/wk (Optifast);	CER: −30% of maintenance needs (individualized)	12 wks	HbA1c, CGM (LBGI, HypoG events), weight, BMI, trunk fat (DEXA), BP, lipids, TDI	No HbA1c/lipid changes; Both groups lost weight: IF −7.0% vs. CER −3.9% at 12 wks; visceral fat ↓ 12.2% vs. 10.1%; sustained weight ↓ only in IF.
Schmidt et al. (2019) Denmark [92]	Crossover	Ns =14; Ne = 10; 44 ± 12 yrs T1D (y) 19 (13–32)	LCD < 100 g/d CHO (isocaloric)	HCD > 250 g/d CHO (isocaloric)	12 wks	HbA1c; DXA; BP; lipids; IM	HbA1c ↔, TIR ns (68.6% vs. 65.3%, *p* = 0.316); LCD ↓ % < 3.9 (1.9% vs. 3.6%, *p* < 0.001), ↓ CV (32.7% vs. 37.5%, *p* = 0.013); weight −1.9 kg vs. +2.7 kg.
Kaur et al. (2020) India [31]	Parallel	Ns/e = 30 (15 T1D and 15 CD); 25.7 * vs. 27.7 * yrs T1D (y) ND	GFD with full gluten elimination, dietitian counselling, and adh. monitoring (tTG-IgA).	Regular gluten-containing ADA meal plan.	12 mo	HbA1c, ID, BMI, frequency of HypoG, bone and biochemical markers	↓ Hypoglycemic episodes/month (3.5 → 2.1; *p* = 0.03); HbA1c ↓ by 0.73% in GFD vs. ↑ by 0.99% in control (*p* = 0.02); ↑ BMI (*p* = 0.002); ns differences in CGM time in HypoG or bone markers; no severe HypoG.
Al-Sari et al. (2021) Denmark [93]	Cross-over	Ns = 14; Ne = 10; 43.6 * yrs; T1D (y) 24.5 ± 13.4 y	LCD (<100 g CHO/d)	HCD (>250 g CHO/d)	12 wks	Lipidomics (sphingomyelins, phosphatidylcholines), BMI, HDL	↑ sphingomyelins and phosphatidylcholines; PPC 35:4 inversely associated with BMI and positively with HDL (*p* < 0.001).
Dimosthenopoulos et al. (2021) Greece [94]	Crossover	Ns/e = 15; F = 67%; 36.1 * yrs T1D (y) 12.4 *	HPD: 20% CHO, 40% PROT, 40% fat; MedDiet: 40% CHO, 25% PROT, 35% fat.	REF: 50% carbohydrates, 20% PROT, 30% fat.	3 wks	GC % time in euglycemic range (TIR 70–140 mg/dL).	HPD—a positive impact on glycaemic control in T1D compared to REF and MedDiet. The HPD reduces time spent in HypoG and lowers GV.
Isaksson et al. (2021) Sweden [95]	Parallel	Ns/e= 159 F= 57.9%; 48.6 (12.0) yrs; T1D (y) 22.3 (11.6)	FBA (food-based, low-GI foods, fish, legumes, nuts, veg, whole grains) or CC	RC (4 standard nurse visits)	12 mo	HbA1c, weight, BP, lipids, hs-CRP, TDI, ACR, mild hypoglycemia, SMBG profiles, diet quality, QoL	HbA1c at 12 mo: no sig. diff. FBA vs. RC (- −0.4 mmol/mol), CC vs. RC (−0.8), FBA vs. CC (+0.4); short-term (3 mo) HbA1c ↓ in CC (−2.9 mmol/mol, *p* = 0.0057) & FBA PP (−3.0, *p* = 0.0171); TG ↓ asix 6 mo CC vs. RC (−0.18 mmol/L, *p* = 0.041); mild hypoglycemia ↑ in FBA vs. RC (+0.39/mo) & FBA vs. CC (+0.35/mo, *p* < 0.001); diet quality ↑ in FBA.
Igudesman et al. (2023) USA [72]	Parallel	Ns/e = 38 (LCD *n* = 16, Look AHEAD *n* = 12; MedDiet *n* = 10); 26.1 (23.6–27.2) yrs; T1D (y) ≥ 1y	HLCD (15–20% CHO) or hypocaloric low-fat Look AHEAD (<30% FAT); MedDiet (not calorie-restricted)	Three-arm comparison (no separate control)	3 mo	HbA1c, CGM: %TBR <70/<54 mg/dL, %TIR 70–180, %TAR 181–250/>250, CV; body fat % (DXA), Weight	↓ HbA1c −0.91% (*p* = 0.005); 58%; Look AHEAD: ↓ HbA1c drop (−0.65%, *p* = 0.027); MedDiet ↑ %TAR (30% vs. 17–18%) Overall: −2.7 kg (*p* < 0.0001).
Isaksson et al. (2024) Sweden [96]	Crossover	Ns= 54; Ne = 50; F = 50%; 48 (22–73) yrs; T1D (y) 22.3 (11.6)	MCH: CHO 30% EI, PROT 20% E, fat 50% EI	Traditional diet: CHO 50% E	4 wks	MG (mmol/L)	MCH diet ↓ MG −0.6 mmol/L vs. traditional; TIR ↑ 4.7%, TAR ↓ 5.9%; no ↑ risk of hypoG or ketoacidosis.
Kahleova et al. (2024) USA [88]	Parallel	VG: Ns = 29; Ne = 18; Age 51.4 (19—79); CON: Ns = 29 vegan group (VG) Ns = 29 17 47.5 (21—72) yrs T1D (y) NR	LF vegan	CON: Diet portion-controlled	12 wks	TD1: HbA1; Total cholesterol, LDL, creatinine nitrogen, CG	LF vegan vs. CON: HbA1c −0.8 vs. −0.6 pp (treatment effect −0.2; 95% CI −0.7 to +0.2; *p* = 0.34); TDI −12.1 vs. −1.4 U/day (effect −10.7; 95% CI −21.3 to −0.2; *p* = 0.046); TC −32.3 vs. −10.9 mg/dL (effect −21.4 mg/dL; 95% CI −35.6 to −7.2; *p* = 0.004); baseline lipids normal.
Kristensen et al. (2024) Denmark [97]	Crossover	Ns/e = 12; F = 33%; 50 (22–70) yrs; T1D (y) 25 (11–52) y.	HF: CHO 19%, fat 62%, PROT 19% (100 g CHO/day) 2) HPD: CHO 19%, fat 57%, protein 24% (100 g CHO/day)	HCD: CHO 48%, fat 33%, PROT 19% (250 g CHO/day)	1 wk per diet, 3 diet periods with 5–35 days washout	HbA1c, ID, body weight, lipids, BP; CGM-based glucose metrics (MG, CV, TIR, TAR, TBR)	HF and HPD ↓ GV vs. HCD (CV: 30.5 ± 6.2%, 30.0 ± 5.5% vs. 34.5 ± 4.1%; *p* < 0.01); HPD ↓ time >10 mmol/L (22.3 ± 11.8%) vs. HF (29.4 ± 12.1%) and HCD (29.5 ± 13.4%); ↑ TIR with HPD vs. HCD (75.8 ± 11.5% vs. 67.5 ± 13.1%, *p* = 0.04); ↓ hypoG events and ↓ total insulin dose with both HF and HPD.

Explanation of abbreviations: *—range not specified; ABC—automated bolus calculator; ACR—albumin-to-creatinine ratio; BMI—body mass index; BP—blood pressure; CC—carbohydrate counting; CER—Continuous energy restriction; CGM—continuous glucose monitoring; CON—control group; CV—coefficient of variation (glucose variability); DEXA/DXA—dual-energy X-ray absorptiometry; eGDR—estimated glucose disposal rate; EI—energy intake; F—female; FBA—food-based approach (diet based on low-glycemic index foods, fish, legumes, nuts, and similar products); FAT—fat; GV—glucose variability; GFD—Gluten-free diet; HbA1c—glycated hemoglobin A1c; HD—habitual diet/high carbohydrate diet; HCD—high carbohydrate diet (>250 g/d); HLDD—hypocaloric Low-carbohydrate diet; HF—high-fat HPD—high protein diet; hs-CRP—high-sensitivity C-reactive protein; HypoG—hypoglycemia; IF—intermittent fasting; IM—insulin management; INT—intervention group; LCD—Low-carbohydrate diet; LF—low fat; LBGI—low blood glucose index; Look AHEAD—Action for Health in Diabetes; MAGE—mean amplitude of glycemic excursions; MCH—moderately carbohydrate diet; MC—multiple daily carbohydrate counting; MedDiet—Mediterranean diet; MG—mean glucose; Ns/Ne—sample size: Ns—number of recruited participants; Ne—final number/completed the study; ns—not significant; pp—percentage points; PROT—Protein; QoL—quality of life; RC—routine care; SMBG—self-monitoring of blood glucose; SpD—special diet; TAR—time above range; TBR—time below range; TDI—total daily insulin dose; TGs—triglycerides; TIR—time in range; U/d—units per day; veg—vegetables; WC—waist circumference; wks—weeks; yrs—years old; ↑ increase; ↓ decrease; ↔ no change; → tendency or direction of change.

#### 3.1.3. Psychosocial and Quality-of-Life Outcomes Associated with Dietary Interventions in Adults with Type 1 Diabetes

This section includes 9 studies evaluating the relationships between diet, behavior, and psychosocial well-being (QoL, FoH, treatment satisfaction, depression/stress, mindfulness). The included studies were predominantly cross-sectional analyses, with occasional non-randomized intervention studies.

The most studied dietary models were the MedDiet (*n* = 4), and healthy eating patterns or diet quality indices (*n* = 4). One interventional study evaluated an LCD, while two studies from Finland assessed habitual dietary patterns. Furthermore, two narrative studies explored broader aspects of eating habits and self-management.

In line with the overall study design, the research was primarily conducted in Europe, specifically in Finland and next, Spain, Greece, and the Netherlands. Individual studies also originated from Iran and the United States.

Full details of the study characteristics, psychological outcomes assessed, dietary assessment methods, and main findings are presented in Table 4.

To conclude, although the available evidence is limited, it suggests that dietary interventions may lead to measurable improvements in QoL, treatment satisfaction, and a reduced FoH in adult patients. However, the scale and durability of these effects are inconsistent based on the available evidence. Compared to metabolic endpoints, the inclusion of psychosocial endpoints in diet studies is limited, highlighting the need for greater integration of behavioral dimensions in nutrition research.

These empirical findings have influenced the evolution of dietary recommendations for adults with T1D, which are discussed in the following Section 3.2 (Evolution and Current Status of Dietary Recommendations).

**Table 4:** The psychosocial determinants of dietary adherence in adults with T1D.

Author, Year, Country	Population	Psychosocial Factors and Measures	Dietary Adherence Measure	Main Results
Ahola et al. (2016) Finland [98]	Ns = 798 Ne = 615; F = 66%; mean age approx. 48 * yrs; T1D (y) approx. 31 y	FoH two self-report items: (1) “Afraid of hypoG” and (2) “Eating ‘just in case’ due to FoH” (FoH = yes to both)	HD via DQ + 19-item FFQ and two 3-day FR (3–6 days total).	FoH → ↑ HbA1c (OR = 1.53, 95%CI 1.09–2.15), ↑ CHO intake (OR = 1.008/g, 95%CI 1.003–1.013), ↓ “high-fat” factor; in women: ↑ SMBG & ↑ EI/CHO intake; no diff. in PA or insulin dosing.
Ahola et al. (2018) Finland [54]	Ns/e = 976; F = 59%; 48 (36–60) yrs. T1D (y) NR	DepS/BDI	Validated DQ + 7-pt FFQ → 7 patterns (Fish&veg, Sweet, Modern, Legumes&veg, Traditional, HF cheese&eggs, Healthy snack); two 3-day FR (6 d); E/macronutrients; SMBG.	12% had DepS (BDI ≥ 16); higher DepS → ↓ E, PROT, fat & CHO; “Fish&veg” & “Traditional” → ↓ BDI; “Sweet” → ↑ BDI; PROT→CHO/fat substitution → ↑ BDI; DepS → ↑ SMBG; HbA1c NS.
Martyn-Nemeth (2019) USA [99]	Ns/e = 30; F = 63%; 30 (20–57) yrs; T1D (y) 16 ± 11 y	FoH; life/work stress; coping strategies; anxiety related to sleep and exercise	No dietary adh. instrument; diet discussed narratively: HypoG management & food use	FoH & chronic stress → ↑ glucose, hypoG avoidance, compensatory eating; work stress ↓ diet attention; some used coping (meal planning, temp basal, social support) but healthy eating was complex.
Granado-Casas et al., 2020, Spain [100]	Ns/e = 258; F = NR; NR ≥ 18 yrs; T1D (y) ≥ 1.	ADDQoL-19 (diabetes-specific QoL) and DTSQ-status (treatment satisfaction)	aMED i aHEI (based on FFQ).	MedDiet adherence ↑ → diabetes-specific QoL ↑; global TS ↔, ale “convenience/flexibility”.
Ahola et al. 2020 Finland [101]	Ns/e = 100; F = 49% 40 (25 −71) yrs T1D (y) NR	PS (Cohen’s 14-item PSS)	Diet score (0–22): fish, veg.; LF milk; veg. oils)	Higher PS → ↓ overall diet score & ↓ adh. to fish, fresh veg. LF milk prod. & veg. oil fats; PS → ↑ mean BG only in lean (BMI < 25 kg/m^2^), not in OW/OB.
Liu et al., 2021, Netherlands [102]	Ns/e = 296; F= 57.8% 47.3 * yrs; T1D (y) 23.6	FFMQ-SF (mindfulness); PHQ-9 (depression); GAD-7; DD-PAID-20 (diabetes distress)	DHD15-index (0–120); “Psychosocial”→ FFMQ-SF (mindfulnes); PHQ-9 (depression); GAD-7; PAID-20 (diabetes distress).	Higher total mindfulness → ↑ diet quality (β = 0.14, *p* = 0.02); “observing” facet also ↑ (β = 0.15, *p* = 0.01).
Turton et al. 2023 # Australia [103]	Ns = 20; Ne = 16; F = 50%; 43 (18–70) yrs T1D (y) ≥ 6 mo	HbA1; TDI; TIR; DQoL; FoH; BMI; creatine kinase	LCD 25–75 g/d) vs. HD (>150 g/day) on HbA1c.	TDC: 214 → 63 g/day (*p* < 0.001); HbA1c: 7.7% → 7.1% (*p* = 0.003); TDI: 65 → 49 U/day (*p* < 0.001); TIR: 59% → 74% (*p* < 0.001); DQoL: ↑ (*p* = 0.015); hypoG freq.: NS; body weight & BMI: ↓ (*p* < 0.025); CK: +32 ± 119 U/L (*p* = 0.008).
Núñez-Baila et al., 2024 Spain [104]	Ns/e = 362; F = 67.4%; 22.8 (18–29) yrs; T1D(y) 11.9 (1–28) y	OSQ; sociodemographic factors; HbA1c; HRQoL	MedDiet Adh. Screener (MEDAS, 0–14; ≥8 = adherent).	Higher MedDiet adh. → ↑ Self-care (β = 0.126, *p* < 0.05) & ↑ Well-being (β = 0.134, *p* < 0.01); higher HbA1c → ↓ Self-care (β = −0.307, *p* < 0.001).
Karipidou et al. 2025 Greece [105]	Ns/e = 192; F = 61%; 42 (34–51) yrs; T1D (y) ≥ 23 (13, 31)	Sleep quality (Athens Insomnia Scale, GC)	MedDiet Score (MLI); PURE Diet Score (PLI).	Better glycaemic control (HbA1c < 7%) → ↑ MLI & PLI (*p* = 0.011; *p* = 0.008); each PLI pt ↑ odds HbA1c < 7% (OR = 1.16, 95%CI 1.01–1.35); ↔ MLI; healthy lifestyle (diet + sleep + activity + non—smoking) → better control.

Explanation of abbreviations: * —range not specified; #—single-arm, non-randomized; ADDQoL—Audit of Diabetes-Dependent Quality of Life; adh.—adherence; BDI—Beck Depression Inventory; BG—blood glucose; BMI—body mass index; CHO—carbohydrates; CK—creatine kinase; DepS—depressive symptomatology; DHD15—Dutch Healthy Diet 2015 index; DQoL—Diabetes Quality of Life; DD—diabetes distress; DDS-17—Diabetes Distress Scale (17 items); DQ—diet questionnaire; E—energy; F—female; FFMQ-SF—Five Facet Mindfulness Questionnaire—Short Form; FFQ—Food Frequency Questionnaire; FoH—fear of hypoglycemia; FR—food record/food recorder; GAD-7—Generalized Anxiety Disorder Scale (7 items); HD—habitual diet; HbA1c—glycated hemoglobin; HF—high-fat; HRQoL—health-related quality of life; INS—insulin dose; LCD—Low-carbohydrate diet; LF—low-fat; MEDAS—Mediterranean Diet Adherence Screener; MedDiet—Mediterranean diet; MLI—Mediterranean Lifestyle Index; NR—not reported; ns—not significant; NS diff./no diff.—no difference; Ns/Ne—sample size: started sample (Ns), effective sample (Ne); OSQ—Oviedo Sleep Questionnaire; OW/OB—overweight/obese; PA—physical activity; PAID-20/DD-PAID-20—Problem Areas in Diabetes (20 items); PLI—PURE Lifestyle Index; PROT—Protein; PSS—Perceived Stress Scale (Cohen’s 14-item); PS—perceived stress; QoL—quality of life; SpD—special diet; SMBG—self-monitoring of blood glucose; SDSCA—Summary of Diabetes Self-Care Activities; T1D—type 1 diabetes; T1D (y)—diabetes duration; TDC—total dietary carbohydrate; TDI—total daily insulin dose; TIR—time in range; U/d—units per day; veg.—vegetables; yrs—years old/age; ↑ increase; ↓ decrease; ↔ no change; → tendency or direction of change.

### 3.2. Evolution and Current Dietary Recommendations for Adults with Type 1 Diabetes

In this study major nutrition guidelines released between 2015 and 2025 were examined regarding their scope, intended population, and principal dietary recommendations for adults with T1D. Most documents endorsed individualized medical nutrition therapy, CHO awareness/education, and the use of CGM-informed adjustments; several provided examples of food-based patterns compatible with glycemic targets. Detailed characteristics are provided in Table 5.

**Table 5:** Energy and macronutrient recommendations for adults with type 1 diabetes across regional and international guidelines.

Guidelines	Energy	Carbohydrate	Fat	Protein	Fiber	REF
ADA (2025)	Management and weight reduction are important, depending on the patient’s needs	45–65% EI; usual ~45% EI (↓ CHO)	<30% EI	15–20% EI	≥14 g/1000 kcal	[2]
EASD (2023)	Individualized	Wide acceptable range; avoid ketogenic VLCD	SFA < 10%, TFA < 1%	10–20% EI (15–20% ≥65 y)	≥35 g/d	[5,7]
ICMR—India	Like the general population	50–55% EI; sucrose <10% E (preferably <5%)	≤30% EI	15–20% EI	≥14 g/1000 kcal	[7]
IDF	At the level of demand	Balanced; emphasis on low-GI	Healthy sources; avoid SFA/trans	Complete; no fixed %	Encourage high fiber; no fixed amount	[1]
Diabetes Canada	Individual requirement	45–60% EI; free sugars <10% E (preferably <5%)	0–35% EI; SFA <9% EI; avoid trans fats	15–20% EI (~1–1.5 g/kg)	≥25–38 g/day	[10]
DDG 2025.	Fully individualized	Flexible; no fixed %	Focus on fat quality (↓ SFA, ↑ MUFA/PUFA)	≥0.8 g/kg/d; ≥1 g/kg/d in older	≥30 g/d	[106]
Japan Diabetes Society	25–35 kcal/kg × activity factor	50–65% EI (DRI); practice: CC	20–30% EI; SFA ≤7% EI	13–20% EI; adjust to goals	≥21 g (M); ≥18 g (F)	[107]
Australian Diabetes Society	Adapted to the patient	~50% EI; portion-based counting	<30% EI; consider glycemia	Protein focus (>40 g with meal if needed)	Depending on lifestyle	[108]
British Dietetic Association (BDA)	Individual requirement (age, sex, activity, goals)	Include CHO at each meal; wholegrains, fruit, veg; avoid sugary drinks	Healthy fats; limit SFA; avoid processed foods	No fixed %; balanced diet (lean meats, pulses, dairy alternatives)	Encourage high-fiber foods; no fixed amount	[9]
Korean Diabetes Association	Individualized	≤55–65% EI (reduce from 65–70%)	<30% EI; limit SFA	15–20% EI	≥20–30 g/d	[109]
Chinese Diabetes Society	25–30 kcal/kg IBW/d	45–60% EI; VLCD not advised	20–35% EI; SFA < 12%, TFA < 2%	15–20% EI	25–36 g/d (12–14 g/1000 kcal; 10–20 g soluble)	[61]

Explanation of abbreviations: ADA—American Diabetes Association; APJCN—Asia Pacific Journal of Clinical Nutrition; CC—Carbohydrate Counting; CHO—Carbohydrate; CGM—Continuous glucose monitoring; DDG—Deutsche Diabetes Gesellschaft (German Diabetes Association); DNSG—Diabetes and Nutrition Study Group; DRIs—Dietary Reference Intakes; EI—Total energy intake; EASD—European Association for the Study of Diabetes; EE—Energy expenditure; GI—Glycaemic index; IBW—Ideal body weight; ISPAD—International Society for Pediatric and Adolescent Diabetes; kcal—Kilocalorie; MUFAs—Monounsaturated fatty acids; PUFAs–Polyunsaturated fatty acids; SFAs—Saturated fatty acids; TFAs—Trans fatty acids; ↑ increase; ↓ decrease.

These limitations underscore the key research gaps that are discussed in the following Section 3.3 (Evidence Gaps), which outlines future directions required to strengthen the scientific foundations of dietary therapy in adult patients.

### 3.3. Evidence Mapping

We constructed an evidence map that arrays dietary intervention types on the X-axis and main outcome categories (HbA1c, insulin dose, glycemic variability, TIR, lipids, weight/BMI, diet quality/adherence, QoL, and safety—hypoglycemia) on the Y-axis. Each bubble represents an aggregated cell defined by intervention type × outcome category; bubble size encodes the cumulative sample size (n) across studies contributing to that cell, while color denotes study design (e.g., RCTs vs. observational/psychosocial studies). This visualization is intended to show where the literature is dense versus sparse and to highlight research gaps across outcomes and diet categories.

**Figure 2:**
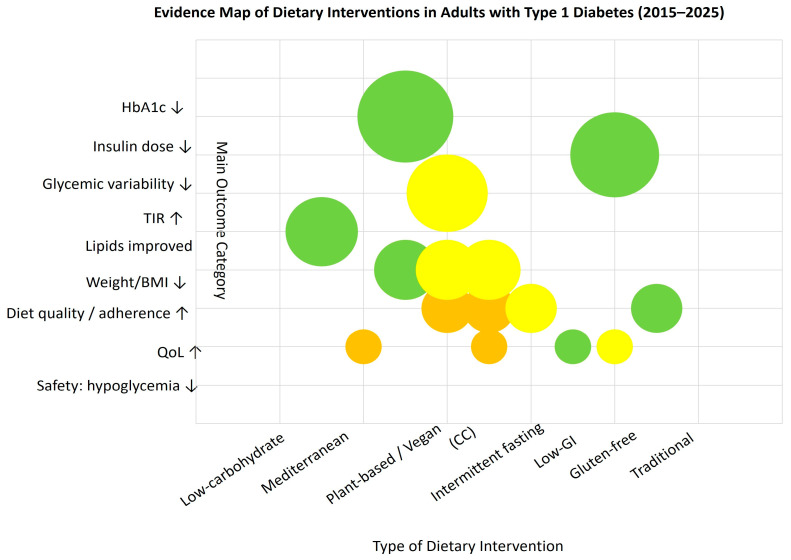
Evidence map of dietary interventions in adults with type 1 diabetes (2015–2025). Each bubble represents an aggregated cell (intervention × outcome); bubble size reflects total sample size, and color indicates study design: green—RCTs, yellow—observational, orange—psychosocial; ↑ increase; ↓ decrease (relative to baseline or control).

The figure is descriptive and does not evaluate individual study quality or estimate effect sizes. Classification rules and aggregation procedures are detailed in the Methods; individual publications are not shown. As shown in Figure 2, most evidence clusters around low-carbohydrate and Mediterranean-type diets, with fewer studies addressing psychosocial or quality-of-life outcomes.

## 4. Discussion

The current scoping review has compiled current data on dietary interventions and eating patterns in adults with type 1 diabetes from observational studies, RCTs, and studies focusing on psychosocial factors. In all studies, an LCD and systematic CC were the most effective strategies for improving GC and reducing insulin requirements [25,72,89,90,103]. In contrast, the MedDiet and plant-based diet were primarily associated with better food quality and overall well-being [76,83,84,90,94]. The wide variety of study designs, intervention durations, and outcome definitions is the primary determinant limiting the comparability of studies, indicating that standard approaches are necessary. Existing studies primarily focus on glycemic outcomes, with HbA1c and descriptive statistics from CGM most frequently assessed [110].

In contrast, the available data concerning cardiometabolic risk factors and behavioral outcomes are comparatively scant and heterogeneous. Meeting these goals will require long-term, well-designed studies that assess both metabolic and patient-reported outcomes. The evidence from such trials will support more effective, individualized nutrition therapy.

### 4.1. Metabolic and Dietary Effects

The reviewed studies collectively explored a broad spectrum of metabolic outcomes, including HbA1c, CGM-based indices, lipid profile, and anthropometric measures. Such comprehensive assessment allows for a multidimensional interpretation of dietary intervention effects in adults with type 1 diabetes.

This synthesis integrates evidence from metabolic, behavioral, and psychosocial domains to provide a comprehensive understanding of dietary interventions in T1D adults. We included 41 studies (18 observational, 14 RCTs, and 9 examining psychosocial factors and nutrition) in T1D adults. Despite differences in design and follow-up, the evidence suggests that diet quality, CHO and protein intake, nutrition education, and technology-enabled self-management are key determinants of GC and metabolic well-being in the study population.

In observational analyses, an LCD increased the likelihood of maintaining HbA1c ≤ 7% by 2.75 (*p* = 0.011), while glycemic control increased this likelihood by 3.27 (*p* = 0.007) [90]. RCTs have confirmed that LCD reduces HbA1c by an average of 0.65% (95% CI, −1.1 to −0.18) [72,89,103]. Across trials, moderate CHO restriction lowered the MG by 0.6 mmol/L and increased TIR by 4.7% (*p* < 0.01); the use of a bolus calculator improved HbA1c by 5 mmol/mol compared to 2 mmol/mol with manual calculations (*p* = 0.033) [25].

In various studies, the reduction in HbA1c ranged from approximately 0.3% to 0.9% [35]. Individual reports from LCD studies also reported reductions of up to 2.9% [64,111]. A total of 11 studies demonstrated statistically significant effects on primary endpoints, most commonly HbA1c and CGM-derived metrics (e.g., TIR/GV) or MC/insulin dose (ID) (*p* < 0.05) [70,71,73,74,77,79,81,85,86,87,112]. Six studies showed no significant differences in the assessed endpoints [48,76,83,84,113], and several did not report *p*-values. In the RCTs subset, five studies showed significant improvement in HbA1c [25,88,89,96,114], and three reported a significant increase in TIR [25,94,96]. Metabolic signals remain broadly consistent across all projects analyzed in this scoping review. Higher fiber and protein intake is associated with lower glucose levels and reduced variability.

Meanwhile, a low-carbohydrate (LC), higher-protein approach reduces the frequency of HypoG [64,92,93,94]. Both earlier studies and those included in this scoping review have confirmed that vegan and Mediterranean interventions result in moderate weight loss and a reduction in insulin dosage of approximately 15–20% [72,88,90]. However, these types of dietary interventions only guarantee limited changes in the lipid profile and blood pressure in the assessed population [64,88,89,91,100]. Other studies have shown that a low-fat vegan diet reduced HbA1c by 0.8%, compared to 0.6% in the control group [88]. In contrast, a Western diet, rich in desserts and saturated fats, is associated with higher HbA1c (OR ≈ 2.5; increase of ~1.0%) [78,85,86]. On the other hand, studies demonstrate that increasing dietary fat causes early postprandial glucose reduction but late hyperglycemia, requiring additional insulin dosing of 6–21% depending on fat content [115].

Mediterranean, DASH, and HEI dietary patterns are associated with lower BMI and waist circumference, lower blood pressure, and better QoL, although not always with lower HbA1c [73,80,100]. Overall, individualized therapy that combines CHO control, education, and technology (CGM and automated insulin delivery) is the operational core of effective nutritional care in T1D [70,71,72,77,82,88,89,95,96,116].

Interventions based on LCD or balanced nutritional strategies (Mediterranean, plant-based) improve metabolic and patient-reported outcomes [83,88,90]. Reduced insulin requirements are the most common and durable effect; however, variability in glycemic responses and potential risks (HypoG, dyslipidemia, micronutrient deficiencies) require individual monitoring [98]. Limited data also suggest potential benefits of a gluten-free diet in individuals with coexisting celiac disease [31].

Taken together, the findings support the following clinical implications. Clinicians can implement LC or Mediterranean dietary approaches under team supervision, with concurrent monitoring of metabolic safety and nutrient adequacy. Utilize a patient-centered plan that centers CC at its core. Clinicians should utilize digital tools (e.g., bolus calculators) and CGM to inform daily treatment decisions. During Ramadan fasting or high-intensity exercise, they should adjust CHO intake and ID accordingly [41,43,117,118].

### 4.2. Comparison and Adaptation of Guidelines

The findings gathered in this scoping review are consistent with previous syntheses. Reviews and meta-analyses indicate that LCDs generally improve HbA1c, although the optimal CHO threshold, intensity of education, and follow-up frequency remain undetermined [25,119,120]. In various studies, CC reduced HbA1c by approximately 0.49–0.64%, while structured education (e.g., Dose Adjustment For Normal Eating—DAFNE) achieved a reduction of approximately 1% after six months [28].

The authors of a 2025 systematic review with meta-analysis demonstrated that high-fiber approaches resulted in a reduction in HbA1c of approximately 0.46%. In addition, they demonstrated that LC or low-glycemic strategies increased TIR by 3.8% (95% CI: 2.2–5.4%) and reduced GV by 3.2% (95% CI: −5.5 to −1.0%) [13]. Due to the involvement of chronic, low-grade inflammation in the pathogenesis of DM, there is still ongoing debate in the literature regarding the consistency of the effects of interventions (or dietary exposures) on HbA1c and inflammatory markers such as hs-CRP [90,95,121]. Small sample sizes, short study duration, and inconsistent reporting of endpoints related to hypoglycemia and QoL continue to limit the certainty of the results, reinforcing calls for longer RCTs with standard endpoints (HbA1c, TIR/TBR, GV, PRO) and consistent definitions of interventions [44,111,119,122].

Recently, guidelines have evolved from rigid target macronutrient ratios to an approach based on individualized MNT. Earlier statements ADA/EASD/ISPAD before 2014) recommended 45–60% EI from CHO, ≤35% from fats (with SFA <10%), 15–20% from protein, and 20–35 g/day of fiber [7,123]. More recent recommendations (ADA 2019–2021; DDG 2025; Korean Diabetes Association 2023; DNSG/EASD 2023) emphasize flexibility, encourage CC, promote ≥35 g/day of fiber, limit SFA to <10% of energy and trans fats to <1%, and discourage the use of VLCD/ketogenic diets [106,109]. The 2022 Chinese MNT guidelines maintain a quantitative approach (45–60% carbohydrates; 20–35% fats, including <12% saturated fats; 15–20% protein) and also advise against VLCDs [61].

There are still gaps in the implementation of these recommendations. In Spain, only 25% of people achieved the target CHO intake, 16% achieved SFA <10%, and 8.9% achieved the recommended fiber intake [124]. Across Europe, the average fiber intake is approximately 8 g/1000 kcal, and SFA intake often exceeds 10% EI [22]. In Ethiopia, adherence to the recommendations reached 44.3% and was associated with nutrition education (OR 2.8; 95% CI: 1.97–5.61) and longer duration of diabetes (OR 2.9; 95% CI: 1.32–5.84), while social and financial barriers hindered adherence [125]. Greater access to CGM and insulin pumps in high-income settings tends to strengthen the impact of dietary strategies, whereas constrained resources elsewhere can blunt these effects [126]. Local food systems and cultural practices, for example, the broader availability of whole grains in parts of Asia, also shape feasibility and adherence [12,60].

### 4.3. Psychosocial and Behavioral

The results of our scoping review confirm a close relationship between the psychosocial context and dietary approaches, as well as glycemic control. Analyses from the Finnish Diabetic Nephropathy Study cohort (FinnDiane) show that FoH correlates with higher HbA1c (OR = 1.53; 95% CI 1.09–2.15) and greater CHO intake (OR per g = 1.008; 95% CI 1.003–1.013) [113]. In addition, sex-specific patterns emerged in self-monitoring and energy intake, with no differences in physical activity or insulin dosing [98].

In a related analysis from the same cohort, approximately 12% screened positive for depressive symptoms (BDI ≥ 16). The “Sweet” dietary pattern was associated with higher BDI, whereas the “Fish & vegetables” and “Traditional” patterns were associated with lower BDI. Depressive symptoms correlated with higher Self-Monitoring of Blood Glucose (SMBG), while HbA1c did not differ [54]. A higher degree of alignment with the Mediterranean dietary pattern was linked to improved glycemic outcomes [101], while higher mindfulness was associated with better diet quality (DHD15; β = 0.14, *p* = 0.02) [102].

Adherence to the MedDiet was associated with higher diabetes-related QoL and, in young adults, with better self-care (β = 0.126, *p* < 0.05), and greater well-being (β = 0.134, *p* < 0.01). In contrast, higher HbA1c correlated with poorer self-care (β = −0.307, *p* < 0.001) [92,112]. In Greece patients, higher scores on the PURE Diet Score and Mediterranean Lifestyle Index, and a set of healthy behaviors, co-occurred with HbA1c < 7% (*p* = 0.008 and *p* = 0.011) [105].

A 12-week, single-arm program with substantial CHO reduction improved TIR (59% → 74%, *p* < 0.001), HbA1c (7.7% → 7.1%, *p* = 0.003), and diabetes-specific QoL (*p* = 0.015) without increasing HypoG [103]. Qualitative evidence shows that FoH and chronic stress promote avoidance behaviors and compensatory eating; work-related stress further undermines dietary goals even when patients employ coping strategies (planning, temporary basal adjustments, social support) [99].

Overall, depression burden, diabetes-related stress, and FoH impair engagement and glycemic outcomes, while self-efficacy, skills, and education support better control [5,52,71,127,128,129,130,131]. These observations are reflected in the ADA/EASD guidelines for routine screening for depression, stress, FoH, and eating disorders, as well as the inclusion of psychosocial care in individualized nutritional therapy [5,132,133]. Most datasets are cross-sectional and use heterogeneous instruments; controlled studies with standardized psychosocial endpoints alongside metabolic outcomes are needed to clarify durability and mechanisms.

Future research should prioritize long-term, adequately powered RCTs that incorporate psychosocial outcomes and technology-supported education. Overall, the evidence underscores the need for integrative nutritional approaches that address not only glycemic control but also cardiovascular and metabolic health in adults living with type 1 diabetes.

### 4.4. Limitations and Research Perspectives

The evidence base we have compiled has several strengths, which we will discuss in more detail below. First and foremost, it focuses on actual dietary behaviors, increasingly utilizes CGM endpoints (TIR, GV), and takes psychosocial dimensions into account [25,70,73,88,89,92,96]. Nevertheless, many studies remain short-term (1–24 weeks) and have small sample sizes, with varying methodological quality.

Furthermore, studies assess dietary self-management inconsistently. They rely mainly on self-reporting tools. Examples of these tools include food diaries, 24-h recalls, and FFQ questionnaires. Rarely do they verify or repeat measurements. Ketogenic interventions rarely verify ketosis. Taken together, the data indicate that many protocols reflect an LCD rather than a strict ketogenic regimen. Heterogeneity in defining interventions and outcomes further weakens the inferences that can be drawn [88,89,90,91,119,134].

However, most studies originate from high-income settings, which limits the generalizability of the results to resource-limited regions [73,85,113,134,135,136].

For these reasons, the standardization of nutritional protocols, the validation of adherence measures, and the extension of the observation period should be the focus of future studies. In addition, it is strongly suggested to adopt a stratified nutrition strategy that incorporates metabolic, psychosocial, and regional determinants of response.

Core outcome sets should include HbA1c, CGM metrics (TIR, TBR, GV), HypoG, ID, lipids, and patient-reported outcomes (QoL, stress).

This scoping review highlights the importance of enhancing the credibility and utility of evidence to improve research outcomes and advance the field. To ensure the highest quality and transparency in future studies, intervention projects must be planned and reported based on international methodological standards. RCTs should adhere to the CONSORT guidelines [137] and the updated SPIRIT 2025 principles [138], which emphasize transparency, data sharing, and active patient involvement in protocol development.

Descriptions of dietary interventions should be prepared in accordance with the TIDieR checklist [139] to facilitate their replication. Methodological rigor should adhere to the principles of Good Clinical Practice (ICH-GCP E6 R2) to ensure reliable and clinically useful results [140].

## 5. Conclusions

Our analysis of the evidence revealed that structured nutritional therapy, which combines a low-carbohydrate or Mediterranean diet with education, carbohydrate counting, and modern technologies used in diabetes treatment, yields the most consistent metabolic and psychosocial benefits in the analyzed patient group.

However, current evidence remains limited, mainly due to the short duration of interventions, small sample sizes, and heterogeneous definitions of eating patterns. There is a lack of representative trials on all continents. Future randomized controlled trials should include more extended follow-up periods and use standardized outcome measures. Consistent assessment of adherence to dietary recommendations is also necessary in populations from different geographic regions. In this regard, psychosocial outcomes should also be taken into consideration. This review highlights the role of individualized and structured nutritional therapy, combined with education and technology. Implementing these combined strategies improves both metabolic control and psychosocial well-being in adults with type 1 diabetes.

## Figures and Tables

**Table 1:** PCC criteria applied in the scoping review on dietary interventions in adults with T1D.

Element	Definition	Extent of Consideration
Population (P)	Adult patients (≥18 years) with type 1 diabetes	No restrictions regarding sex, diabetes duration, insulin regimen, or presence of comorbidities. Both younger (20–40 years) and older (≥40 years) adult groups were considered.
Concept (C)	Dietary interventions and their impact on the health of patients with T1D	Evaluation of dietary patterns (MedDiet, DASH, LCH, ketogenic diet, low glycaemic index, vegetarian, vegan, plant-based). Inclusion of dietary modification strategies (e.g., CC, reduction in simple sugars, fiber increase, fasting protocols). Outcomes of interest include metabolic GV, lipid profile, BMI, clinical (hypoG, insulin dose, complications), and psychosocial/behavioral outcomes (QoL, adherence, DD, FoH, eating disorders).
Context (C)	Geographical, cultural, and healthcare system settings related to T1D management	International perspective: recommendations of diabetes societies (ADA, EASD, IDF, Diabetes Canada, Diabetes UK, NDSS Australia, Asian and African Societies). Consideration of healthcare access (dietitian availability, use of technology such as CGM, apps, bolus calculators), socio-economic barriers, regional food availability, and cultural dietary patterns.

## Data Availability

Not applicable.

## Supplementary Materials

The following supporting information can be downloaded at: https://www.mdpi.com/article/10.3390/nu17213349/s1, Table S1: Operational definitions of diets (extracted as reported). Table S2: Carbohydrate exposure categories used for synthesis.

## Abbreviations

The following abbreviations are used in this manuscript:

ADA	American Diabetes Association
BMI	Body Mass Index
CC	Carbohydrate Counting
CGM	Continuous Glucose Monitoring
CHO	Carbohydrate
DAFNE	Dose Adjustment For Normal Eating
DASH	Dietary Approaches to Stop Hypertension
DD	Diabetes Distress
DDG	Deutsche Diabetes Gesellschaft (German Diabetes Association);
DM	Diabetes Mellitus
DNSG	Diabetes and Nutrition Study Group
EASD	European Association for the Study of Diabetes
EI	Energy Intake
FFQ	Food Frequency Questionnaire
FinnDiane	Finnish Diabetic Nephropathy Study cohort
GC	Glycemic Control
GV	glycemic variability
HbA1c	Glycated Hemoglobin
HypoG	Hypoglycemia
ICH-GCP E6(R2)	International Council for Harmonization—Good Clinical Practice, Guideline E6 (R2)
ID	Insulin Dose
IDF	International Diabetes Federation
IDMPS	International Diabetes Management Practices Study
IF	Intermittent fasting
LC	Low carbohydrate
LCD	Low-carbohydrate diet
LADA	Latent Autoimmune Diabetes
MedDiet	Mediterranean Diet
MG	Mean Glucose
MNT	Medical Nutrition Therapy
NDSS	National Diabetes Services Scheme
PA	Physical Activity
PRO	Patient-Reported Outcomes
QoL	Quality of Life
RCTs	Randomized Controlled Trials
SD	Standard Deviation
SMBG	Self-Monitoring of Blood Glucose
T1D	Type 1 diabetes
T1D adults	Adults with type 1 diabetes
TDI	Total daily insulin dose
TIDieR	Template for Intervention Description and Replication
TIR	Time in Range
VLCD	Very Low-carbohydrate diet
