# Supplementary material for: Dietary Interventions for Adults with Type 1 Diabetes: Clinical Outcomes, Guideline Alignment, and Research Gaps—A Scoping Review
Source: Nutrients. 2025 Oct 24;17(21):3349. doi: 10.3390/nu17213349 (PMC12609816; doi:10.3390/nu17213349)
Supplement: Supplementary file 1 [file nutrients-17-03349-s001.zip › nutrients-3877237-supplementary.pdf]

Table S1. Operational definitions of diets (extracted as reported).

Diet	Abbreviation	Operational definition	Comparator (if specified)	Ref.
Low-carbohydrate	LCD	Very-low ≤50 g CHO/d (isocaloric) or low <100 g CHO/d (isocaloric)	High-CHO ≥250 g CHO/d	[64,93,94]
Moderate-Carbohydrate	MCH	~30%E CHO; ~20%EI protein; ~50%EI fat; food base: whole grains, vegetables, unsaturated oils, nuts	Traditional/higher-CHO	[97]
High-protein	HPD	~ 40% protein; ~20% CHO; ~40% fat	MedDiet: 40% CHO, 25% protein, 35% fat; REF: 50% CHO, 20% protein, 30% fat	[95]
High-fat	HF	~62% fat; 19% CHO; 19% protein (100 g CHO/d) + matched-CHO HP arm (19% CHO, 57% fat, 24% protein)	HCD: 48% CHO	[98]
Mediterranean	MedDiet	Emphasis: olive oil, fish, legumes, nuts, vegetables; used as intervention or adherence indices (aMED/MEDAS)	—	[73,84,91,97,105]
Low-fat vegan	LF vegan	Vegan, low-fat pattern	Portion-controlled or low-fat comparator	[89]
Food-based approach	FBA	Education: low-GI foods, fish, legumes, nuts, vegetables, whole grains	CC or routine care	[96]
Intermittent fasting	IF	~600 kcal/d, 2 day/week (non-consecutive)	Continuous energy restriction (approx.−30% needs)	[92]
Gluten-free diet	GFD	Complete gluten elimination; dietitian support; serologic adherence monitoring (anti–tTG-IgA)	Standard ADA meal plan (T1D with subclinical CD)	[31]
Carbohydrate Counting	(CC / advanced CC)	Structured counting education; with/without automated bolus calculator (ABC) vs manual calculations (MC); CC (timing, tools)	ABC vs MC; routine/pragmatic	[25,72]
High CHO	(HCD)	-	HCD 48% CHO	[64,93,94]

Explanation of abbreviations: %E or %EI – percentage of energy intake; CHO – carbohydrates; CC – carbohydrate counting; REF – reference diet; CD – celiac disease.

Table S2. Carbohydrate exposure categories used for synthesis.

Category	Threshold / unit	Notes	Ref.
Very-low / Low CHO	≤50 g/d or <100 g/d	Author-stated; isocaloric	[64,93,94]
Moderate CHO (MCH)	~30%EI CHO	~20%EI protein; ~50%EI fat	[97]
High CHO (HCD)	≥250 g/d	Comparator in OBS/RCT	[64,93,94]

Explanation of abbreviations: OBS – observational studies; %EI - percentage of energy intake; CHO – carbohydrates; MCH – moderate-carbohydrate diet; HCD – high-carbohydrate diet.
